# The Brain in Relation to the Mind

**Published:** 1855-07-01

**Authors:** 


### THE JOURNAL

OF

### PSYCHOLOGICAL MEDICINE

AND

### MENTAL PATHOLOGY.

JULY 1, 1855.

### Art. I.—THE BRAIN IN RELATION TO THE MIND.

It is obvious that physiological psychology cannot be expected to attain that scientific definitiveness characteristic of the more physical departments of philosophical investigation. The relations between the mind and the brain can probably be never determined with scientific precision, the obstacles to such a result being very conceivable. Whilst our researches into purely physical conditions educe facts and circumstances that strike observers with a certain exactness and uniformity, the phenomena of mind—of *consciousness*—are very often but imperfectly apprehended; and even when these latter are sufficiently distinct and clear to admit of notation and record, their significance, in the estimation of inquirers, is by no means uniform. Thus, whilst science in general, including the inferior branches of physiology, has of late years progressed with giant strides, a physiology of brain and mind that commands universal acquiescence has not nearly been attained. In order to realize a system of analytical psychology that should stand in obvious relation with a doctrine of the cerebral functions, we ought to be able to note the varying phases of consciousness in their outward manifestations with some such readiness and facility as that with which we estimate physical conditions. Were this within our power, we might go far to the accomplishment of a truly scientific psychology, based upon our knowledge of the structure and offices of the brain. The inevitable want of an objective standard to measure the value of mental facts, causes them to be devoid of satisfactory comparability: hence, psychical phenomena admit of no perfect system of classification; and, with still less exactitude, can they be compared with physical facts and conditions. Yet scientific induction demands complete and obvious recognition of the comparable worth of all the circumstances that constitute the premises. Again, most of the materials obtainable for establishing conclusions regarding mental phenomena, are made up of certain outward manifestations that do not always suggest a very clear or unmistakeable interpretation. We note the external facts of consciousness in the several grades of animal life, from the lowest creatures up to man; but, in determining their significance, we have to speculate and to reason mainly from the analogies gained by introspection of ourselves. Moreover, in tracing the sequence of phenomena that characterize cerebro-mental action, we have ever to pause upon attaining the last *change in molecular disposition* that causally precedes *inchoate consciousness*. Between the line that bounds the ultimate physical condition and that which borders the primary psychical state, there is an inestimable chasm. The connecting link, indeed, between matter and mind must always remain, as it is, inscrutable to scientific investigation.

And yet these abstract difficulties, inherent in the subject, have not prevented inquisitive minds in all ages from hazarding speculations concerning the relations of psychical phenomena to the physical organization. In later times, indeed, conclusions have been attained with regard to this matter, that carry with them almost every stamp of authenticity short of actual demonstration. In a very early stage of physiological inquiry, it was considered that the brain and nerves had some special connexion with the manifestations of conscious life; and, in modern times, this, as a general proposition, takes rank as scientific truth. The special functions of the spinal cord and of particular nerves were partly anticipated, long before doctrines upon the subject were sustained by demonstration. The idea that the separate ganglia of the sympathetic nervous system were independent sources of power, suggested itself to Dr. Johnson, of Birmingham, about the middle of the last century, prior to Bichat's advancement of a similar hypothesis. And, as is well known, the researches of Gall, early in the present century, brought prominently before physiologists a theory, in principle now generally admitted, to the effect that the encephalon is not a simple organ, but that particular portions subserve different mental functions—a principle the correctness of which is hardly to be doubted, whatever be the errors and exaggerations of phrenologists. But, however just may have been many of the anticipations that prevailed in the early part of the present century, or at epochs anterior to it, very few of the general notions upon the brain and nervous system could be maintained as scientific induction. It is almost altogether of late years that patient and persevering observation, and ingenious experiments, have been systematically applied to obtain results that partake very largely of philosophical accuracy, even when they do not entirely fulfil its imperious requirements. The researches of Bell, Magendie, and Bellingeri, demonstrated the anatomical distinctness of the motor and sentient nerves. Marshall Hall showed by experiment and pathological facts, that the spinal cord is a source of nervous power, independent of the brain, and urged, by convincing reasons, that its influence in the production of muscular movement was exercised without any necessarily attendant consciousness. Numerous facts and observations, particularly the experiments of Axmann, of Berlin, have rendered it probable in the highest degree, that the sympathetic nervous system presides over motions involved in the processes of circulation, nutrition, and secretion. With respect to these inquiries concerning the functions of the nervous system, our knowledge has become considerable, and in many respects exact. Even in those cases wherein the results cannot be absolutely maintained as positive, very promising researches are continually going on, so that our expectations of the future of neurology are of the brightest. But when we come to the encephalon—to those masses of nervous substance surmounting the spinal cord, and enclosed within the cranium—our deficiencies and shortcomings become more apparent. Certain general propositions can be maintained; but, when we would advance to particulars, rational hypothesis must be made to supply the defects of theory, if we are disposed to systematize our opinions and views. We can show by numerous facts and solid argument that some of the structures forming the base of the encephalon constitute seats of sensation and sources of motion, but by rigorous processes of induction we can *prove* little more. When the higher phenomena of consciousness are considered, and when we would establish a connexion of these with the physiological action of parts within the head, the nature of our evidence exhibits a comparative weakness. Certain doctrines now current upon this subject are most probably true, but the testimony sustaining them is of a somewhat different character to that by which the functions of the spinal cord and particular nerves have been made out. Our evidence is less direct; it is circumstantial; and it carries conviction, rather by its cumulative force, than by any immediate demonstration. We appeal to the results of mutilation, to pathological facts, and to comparative anatomy; we note the phenomena of embryonic development, and observe the variation in cranial forms as indicative to some extent of cerebral magnitude and configuration; and, from these several sources of investigation, we arrive at conclusions concerning the physiology of the brain that, in many respects, are but little short of scientific certainty. But when we pass from the general operations of mind and come to such as are special, and attempt to arrange the phenomena in categories,—when we would make out a distinct relation between particular mental faculties and portions of the cerebral structure,—when, in a few words, we attempt the establishment of some complete physiological psychology, it is then that we discover the insecurity of our footing; an insecurity, most likely, that will never be altogether obviated, on account of the inherent difficulties of the subject.

Up to a certain point, however, undoubted advances have been made in this direction. *Some* views of the correlation of psychology and physiology can be shown, having higher pretensions than mere hypothesis and verbal subtlety. In regarding the physiology of the brain and nervous system in its totality, we may probably analyse and sum up our actual knowledge, and the most generally received opinions, very briefly as follows:—The notion propounded during the last century, that the sympathetic ganglia constituted independent sources of nervous power, has led, by gradually ascending generalisation, to the conviction, now all but universal, that the grey tissue of all the nervous masses—the vesicular neurine—is identical in its general character with the structures long denominated ganglia, not only in the fact of its being of vesicular composition, but also in that of its being the primary seat of *functional change*, the influence of which is *conducted* from part to part by the white fibrous substance; the nerve-trunks thus constituting internuncial cords simply. All the sources of our knowledge contribute more or less to the corroboration of this view. Hence the term ganglion is at this time applied, not only to those smaller spheroidal masses always recognised as ganglia, but also to those larger accumulations of vesicular neurine within the cranium, and to those tracts of the same substance pervading the interior of the medulla spinalis. The sympathetic system itself is probably the most simple in its functions, as it, or its presumed analogue, is the most universally found in the various forms of animal organization; its office being apparently to participate, as before observed, in the maintenance of organic life. In this view, consciousness can have no necessary place in its exercise. The vesicular neurine which is continuous throughout the length of the spinal cord and constitutes the analogue of the ventral ganglia of the articulata, is virtually demonstrated to be for the conservation of the animal fabric, by its subservience to respiration, by governance of the orifices of egress and ingress, and by its contribution to the integrity of some other processes, the purposes of which are mainly conservative. Its function is called into exercise by excitation of the peripheral terminations of nerves that communicate with it, or by influences that operate more immediately. No development of consciousness attends the proper action of the ganglionic masses within the spinal column. The first indications of conscious life show themselves coincidently with the nerves and ganglia of the external senses—of smell, taste, hearing, sight, and touch; these senses are obviously associated with collections of vesicular neurine which are situated above the spinal cord, and which, in the higher classes of animals, are protected by the bones of the skull. The sensory ganglia are, by white nerve-fibres, in direct communication with vesicular neurine expanded on the surfaces forming the special regions of the particular kinds of sensibility. Upon these latter the fitting impressions are made, and upon the extension of their influence to the encephalic centres, consciousness of subjective change—sensation—becomes awakened. But at this very point—that at which the correlation of psychology and physiology begins—the demonstrability of prevalent doctrines becomes less complete. Uncertainty to some extent exists thus upon the very threshold. We have even no sure knowledge as to which are the ganglionic centres of touch—the most simple and universal of all sensibilities. Although concerning the ganglia of smell, sight, and hearing we have some reasonable assurance, there is not that fulness of evidence which obtains in many other departments of physiology. The encephalic centre of taste is altogether undetermined. Sensations, in the first instance, determine simple perceptions; and these, as ideas, constitute the elements of thought and fancy. These more complex and varied phases of consciousness are accomplished, it is now very generally believed, through the instrumentality of the vesicular neurine investing the cerebral hemispheres, and hence denominated the hemispherical ganglia. This opinion, though essentially hypothetical, rests upon many substantial grounds, as it accords with the best established facts, alike of general physiology, comparative anatomy, and pathology. Emotional sensibility, and the instinctive appetites, are supposed to have an encephalic locality somewhere among the ganglionic masses situated below the cerebrum proper. And it is commonly thought that harmony in the action of muscles when movement, the result of mental activity, ensues, is secured by the physiological agency of the cerebellum.

This recapitulation of current doctrines of physiology in relation to psychology, comprises views that future investigations may very considerably modify, or altogether set aside. However well supported many of them may appear to be by facts from all sources, they rest upon inadequate foundations, if we would deal with them as with indisputable propositions. By continued researches, they may be made most probably to look much more like truth than even they do at present. It seems to us, however, that with respect to the higher departments of psycho-physiology, complete scientific accuracy is, in the nature of things, not to be anticipated.

And yet the pages of this Journal for many years testify that we would not discourage investigation of this difficult subject, nor attempt to run down theories however incomplete, if rational in themselves and apparently accordant with our well-established knowledge. There is a legitimate and a practical good in reasonable hypothesis; it stimulates inquiry, it fixes the attention and aids the memory in storing up facts, and, more than all, it causes systematic reflection. Of course we speak of its just use, not of the abuse.

Metaphysical speculations regarding individual faculties of the mind and the genesis of mental capacity and power and physiological notions concerning separate cerebral regions for distinct modes of mental action, have often been advanced by ingenious persons, and then been pursued by zealous scholars with keen and earnest partisanship. And however much in advance of all inductive philosophy, apostles and disciples in the ardour of novelty may have gone, useful results to practical science have almost always followed in some degree. The phrenological speculations in particular, having had much plausible foundation, have certainly exerted a beneficial influence upon moral and physical education, and also upon the curative management of abnormal states of the brain and nervous system.

These somewhat desultory remarks have been called forth by the perusal of a work by the distinguished Neurologist, Mr. Swan.* And although we should have had great pleasure in receiving and acknowledging enlightenment from so respected a quarter, we are bound, in the honest performance of our critical duty, to express the great disappointment with which we have studied its contents. We expected—we had an undoubted right to expect, in a book professing to elucidate the Relations between the Mind and the Brain,—that the writer would at least have availed himself of all the discoveries, and of all the best supported opinions, and of all the most truth-resembling hypotheses, that have lately been propounded concerning the brain and the nervous system; and we expected that an attempt would have been made, to exhibit some correspondence between these and the most rational speculations regarding the mental operations. But we find nothing of the kind. We notice in Mr. Swan's volume no reference to any of the advances made in cerebral and nervous physiology since the era of Sir Charles Bell. Mr. Swan, indeed, is in these respects a veritable Rip Van Winkle. The entire foundation in physiology of his various speculations resolves itself into a recognition of a nervous tract for sensation, and one for the several kinds of motion; each communicating with the brain, which in this discussion Mr. Swan somewhat

* "The Brain in Relation to the Mind." By Joseph Swan. 8vo, pp. 113. London: Longmans. 1854.

quaintly denominates the "sensory," scrupulously avoiding the Latin term in common use, *sensorium*. The mental philosophy which our author adopts is very much of the sensational school: sense-impressions are transformed into ideas and thought, in the "sensory;" and this latter reacts upon the system and the outer world through the voluntary motor tract; habit and exercise of mind, in particular modes, very much increasing the correlative power and capacity. We have no recognition of the reflex function of the spinal cord; none of the physiological distinctness of the grey and white bundles of nerve-tissue; we have no notice bestowed upon the modern doctrine, that the hemispherical ganglia are especially concerned in the manifestation of intelligence; none of the view, that the ganglia situated below the cerebral convolutions constitute the organic seat of emotion and sensation, having their distinct and proper reactions upon the muscular system. And, throughout the work, we look in vain for mention of any but the most obvious and commonplace speculations into which psychologists are accustomed to enter. We have neither original nor adopted analysis of the mental faculties; no account of the progressive development of psychical capability, coincidently with advancing perfection of the brain and nervous system. We have withal a very diffuse and obscure style, so involved and complicated as occasionally to produce absolute unintelligibility. Moreover, we have no proper distinction drawn between fact and hypothesis, none between scientific induction and simple opinion. And throughout the volume, indeed, a singular inaccuracy, both of thought and expression, is constantly met with. This is somewhat severe criticism, but the citations we subjoin will be found amply to justify it.

Mr. Swan's Introduction opens as follows:—

"All sensations or feelings pass from one or other of the organs of sense by their respective nerves to the sensory. Some of them are for temporary purposes, and fleeting, so as to become almost as much effaced as if they had never been received.

"The large mass of white fibres tending from the convolutions to the striated body, and thence to the crus of the brain, and the pyramidal body from which all the voluntary nerves arise, constitute by far the largest portion of the entire brain. These fibres of the voluntary tract have a capability of activity, not amounting to motion like that of the muscles, but on being excited possess an energetic or tonic power; so that when a letter or a man's face is transferred to them from the eye, they can change their negative quality into a more positive one, so as to receive the image as a correct miniature, and then conduct it by continuous fibres to join those about to pass through the striated body and crus of the brain to the nerves and muscles of the tongue for speaking, or through the spinal cord and nerves to the muscles of the hand for writing, drawing, or other mechanical device.

"All impulses received from the organs of sense, which are to be accepted for constituting knowledge, are impressed by one or more fibres of the voluntary tract on the sensory conjointly with the mind, and by one or several repetitions are made permanent. All knowledge purely mental is accepted by, and becomes subjected to, the mental faculties, and may at any time be conveyed back from the mind through the voluntary tract and muscles employed in the tongue for speech, and in those of the hand for writing."

The above passages comprehend in germ the whole of our author's doctrines and speculations. The limited physiology, and the obsolete method of applying it, we need not point out; it is too patent. The faults of style, moreover, of which we have spoken, are abundantly exemplified throughout the quotation.

After the Introduction, comes a chapter "On the Gradual Mode of Development of the Faculties of the Mind;" in which, however, we fail to detect the enunciation of a single principle beyond that which is comprised in the fact, that the mental powers evolve themselves by degrees, and become strengthened by exercise. We discover neither novelty in the mode of exposition, nor anything new or striking in illustration; on the other hand, we observe what we deem to be both confusion of thought and inaccuracy of expression. "Every day," says Mr. Swan, "furnishes fresh information," and "this is compared with the preceding results of thinking, and the stock of knowledge becomes enlarged and *corrected*." This of course is true enough, though rather commonplace, and not very well stated; but how are we to deal with the following propositions immediately succeeding the passages just cited?—"A great portion of it (knowledge) may remain fixed in the brain, but the result or meaning is preserved in the mind." Now we can understand that certain material changes which accompany the ingress of knowledge to the mind, may leave their traces in the brain; but how *knowledge* can take up its abode in the cerebral structure, is something entirely past our comprehension. For, let it be observed, Mr. Swan refers to the brain as distinct from the mind, to which latter, indeed, he assigns another office in the same process:—"the result or meaning (of knowledge) is preserved in the mind." Passing by the erroneous employment of *result* and *meaning* in the same sense, let us ask what, as men of science or philosophers, we can predicate of the mind and its operations in contra-distinction to the brain and its functions?

"Memory," says Mr. Swan, "is an *active* condition of the mind and brain, which allows a review or return of the knowledge that has been previously received." But, notoriously, memory is just as often a passive as an active process; *reminiscence* occurs spontaneously and without any active effort, whilst *recollection* demands voluntary exertion.

Indeed, our author's own phraseology clearly suggests the passive character of memory in some of its manifestations; for instance, it "*allows* a return of the knowledge," &c. We do not refer to these points in a spirit of hypercriticism, but in justification of our statement, that Mr. Swan's style was obscure and inaccurate.

We have failed to grasp the meaning of the passage cited below; it undoubtedly approaches *transcendentalism*—a pursuit for which our author, we presume, would claim no special vocation or aptitude. The subjoined extract is from the second chapter, on "Letters and Words as Expressions of the Mind."

"No idea of a spiritual nature can be conveyed to the memory except it be bounded by an outline, and this can be only a nominal representative of it; anything spiritual to be seen must be either brighter or darker than light, that the light may be an outline to the spirit, or that a darker colour of the spirit may be an outline to the light. It is only by similar comparisons that the mind can form an idea of spiritual elements, and therefore it must receive light, bounded by lines, for words, or other symbols."

We have twice very carefully read the above passage; but we doubt if any degree of attention would enable us to paraphrase it, however anxious we might be to communicate to our readers the thought which underlies the phraseology.

The chapter on "Speech, Writing, and Calculation, as Expressions of the Mind," commences with this passage:—"The first instruction enters by the ears, and is spoken by the tongue and lips." Does this proposition correctly express the fact? We think rather that the mind receives its first instruction through the senses of sight and touch, and that the auditory sense is probably the latest that becomes fitted for *informing* the infant capacity. The primary results, moreover, are outwardly manifested in gesture and intonation, long before the tongue and lips subserve articulate speech. Again, our author says,—"Words could not have entered the mind of any one without an effort of the will, through the voluntary tract." To say nothing of the awkwardness of expression involved in mention of the "voluntary tract" in such context, we must demur to the whole statement. It is common experience that both words and their significance frequently enter the mind, without any attention or recognisable voluntary effort. We accidentally hear—not actively listen to—the utterance of some collocation of words; the attention is engaged with thoughts alien to their subject; at the moment when spoken, the words have struck the sense simply as sounds; yet, in a few seconds, it may be minutes, the meaning suddenly breaks upon the intelligence,—and that, too, without any sort of effort of the will.

Another chapter is on "Various Arts as Expressions of the Mind," and it abounds with those faults and blemishes which we have stated to characterize the whole of the work now before us. We submit the following as an example,—"A good painting . . . shows the mind of the artist, by which it was completed through the hand. The eye and will might have directed the muscles to give form and colouring, but the mind of the artist must in the good picture have been co-operating with the will to have produced the mental meanings of the various characters introduced into it." What conception can we rightly form of the will separately from the mind? Is not the will its highest expression in reference to action? A writer who shall speak of the mind co-operating with the will, is obviously out of his depth.

In the present chapter, we have exhibited to us a very extraordinary mode of settling a philosophical difficulty. Mr. Swan furnishes us with what he deems to be a scientific explanation of that remarkable instinct in bees, which leads them uniformly to construct their cells in the hexagonal fashion. The solution of this problem is as follows:—

"The bee and wasp have been considered as possessing correct mathematical ideas in making their cells hexagonal; but their compound eyes are divided by hexagonal marks; and as the motions of the muscles of animals are directed very much by the mode of admission of light, the shape of the cells may be in accordance with that of the surface of the eyes. In all imitations of objects the muscles take a form of action from an organ of sense—the pattern is received by the eye, and thence conveyed to the brain, and having produced in this a precise impression of its form, the action of the muscles is modelled so as to continue its representation. The images of external objects always falling on the brain of the bee or wasp through the hexagonal divisions of the surface of the eye in viewing near objects, produce such habitual motions of the muscles as constitute unconsciously the hexagonal form of cells; and thus one of the faculties of instinct is accounted for, and the cause of the mathematical exactness explained."

Condillac and his school taught the *sensational* philosophy, arguing that the mind is purely passive, and formed in its faculties by agencies from without; but the above reasoning seems to furnish a corresponding application of *mechanical* philosophy, in the explanation of vital and mental phenomena. We are surprised that Mr. Swan should have propounded his theory so confidently as he appears to have done. Many living creatures construct instinctively; young infants will often do so, long before their actions are guided by knowledge and reason; but there are no facts to show that correspondence obtains between the configuration of their eyes and the particular forms which, by preference, they delineate. But we should regard it as a very unnecessary consumption of the reader's time and patience, to enter upon the serious confutation of so far-fetched an hypothesis.

In a chapter on "Different Conditions of the Brain for Co-operating with the Mind," we find that our author ranks with those who maintain that the natural differences of mental capacity are but little, if any. At this we are rather surprised, as the question is one not difficult to be determined by observation, and it is one thoroughly practical; and Mr. Swan, we should suppose, is much happier in dealing with simple matters of fact than with speculative topics. We suppose, however, that, whilst writing his book, he allowed himself to be carried, like so many others, wherever the course of his temporary hypotheses led him. We cite the following:—

"Every fresh impulse requires one or more fresh fibres of the voluntary tract for its perfect reception, and thus so large a brain is necessary for the almost unbounded extent of the powers of the intellect of man; therefore, however hard he may work, and however much knowledge he may acquire, there is always room left for further stocks of information. He can occupy as much of his brain, however, as he pleases, and according to the degree of his industry, or idleness, will be his intellectual progress, so that the extent of his attainments rests with himself."

And the author, in the subjoined passage, still more explicitly evinces his *levelling* tendencies:—

"With respect to the attainments of different persons, there is the utmost variety: ignorance is usually attributed to weakness of memory; it is, however, probable that if proper pains had been taken to impress information on the mind and brain in early youth, there would not have been those occasions for showing such weakness. It would not then be decided that the original powers are much greater in one than in another, as to the quantity of learning capable of being introduced and retained by every person of ordinary powers, if proper methods are used."

We are not sorry that we are approaching our limits, and that we must arrest our pen. We had marked several passages in other parts of the work for citation and comment, but our space is consumed. What we have already given, will justify the unfavourable judgment of Mr. Swan's performance, which we have felt it to be our duty to declare. We know not whether our criticism will meet the eye of Mr. Swan; or, if so, whether he will care for it. But this we know, that to have reviewed anything from so respectable an author otherwise than with commendation, has been to us the occasion of unmitigated regret. For almost a quarter of a century, we have held Mr. Swan in the greatest honour; his admirable plates of the nervous system, drawn, we believe, from his own dissections, have often refreshed our memory when knowledge has been fading, and when the actual subject has been unattainable. We think that our author in deciding to engage himself in discussions of psychology and cerebral physiology, has simply made a mistake,—one, however, that many able men have made before him. Any one who, in the latter half of his life, undertakes studies and researches totally foreign to early pursuits and habits of thought, is all but sure to fail. Sir Isaac Newton won for himself imperishable laurels in the fields of mathematics and natural philosophy, but failed entirely when, in his later years, he took to Biblical exegesis and the interpretation of prophecy. Sir Humphrey Davy achieved renown as a philosophical-minded chemist; when, however, his ambition led him to aspire to a ball-room reputation, we have read that he did not attain to a respectable mediocrity. Mr. Swan has shown himself for long years to have been a most able and industrious worker upon the *anatomy* of the brain and nervous system ; he has clearly failed in his attempts to elucidate and improve the *physiology*. As before observed, very few men can successfully enter upon new pursuits in advanced life ; mental adaptiveness for particular studies must be established and secured in younger days to attain success ; and Mr. Swan will probably acquit us of disrespect to him, if we adduce, in reference to himself, the sentence with which he concludes his fifth chapter. " It is most probable that unless a person is gradually educated from his youth, he will not attain to excellence either in learning or the arts ; and there are great difficulties in changing his position and leaving off an accustomed business, which depended on manual dexterity."

